# Crystal structures of 2-(4-nitro­phen­yl)-3-phenyl-2,3-di­hydro-4*H*-1,3-benzo­thia­zin-4-one and 2-(2-nitro­phen­yl)-3-phenyl-2,3-di­hydro-4*H*-1,3-benzo­thia­zin-4-one

**DOI:** 10.1107/S2056989015004545

**Published:** 2015-03-25

**Authors:** Hemant Yennawar, Aaron S. Cali, Yiwen Xie, Lee J. Silverberg

**Affiliations:** a Department of Chemistry, Pennsylvania State University, University Park, PA 16802, USA; b Pennsylvania State University, Schuylkill Campus, 200 University Drive, Schuylkill Haven, PA 17972, USA

**Keywords:** crystal structure, benzo­thia­zine, screw-boat pucker, nitro-group inter­actions, hydrogen bonding, C—H⋯π inter­actions

## Abstract

The crystal structures are reported of the isomeric compounds 2-(4-nitro­phen­yl)-3-phenyl-2,3-di­hydro-4*H*-1,3-benzo­thia­zin-4-one, (I), and 2-(2-nitro­phen­yl)-3-phenyl-2,3-di­hydro-4*H*-1,3-benzo­thia­zin-4-one, (II), both C_20_H_14_N_2_O_3_S, being the *para*-nitro and *ortho*-nitro forms, respectively, the *meta*-form of which is known [Yennawar *et al.* (2013). *Acta Cryst.* E**69**, o1679]. The six-membered thia­zone ring fused with a benzene ring displays a screw-boat conformation with a total puckering amplitude of 0.627 (1) Å in (I), and a near screw-boat conformation with a total puckering amplitude of 0.600 (1) Å in (II). The dihedral angles between the planes of the substituent nitrophenyl and phenyl and rings with the benzene ring of the parent benzo­thia­zone moiety are 75.93 (5) and 82.61 (5)° [in (I)], and 76.79 (6) and 71.66 (6)° [in (II)]. Weak inter­molecular C—H⋯O hydrogen-bonding inter­actions between aromatic H-atom donors and both a nitro-O atom and a thia­zone O-atom acceptor in (I) and a thia­zone O atom in (II) are present, forming in (I) a centrosymmetric 22-membered cyclic dimer which is extended through a similar inversion-related 14-membered cyclic hydrogen-bonding association into a zigzag chain structure extending along *c*. In (II), a single inter­molecular C—H⋯O hydrogen bond gives a chain structure extending along *b*. In addition, weak C—H⋯π inter­actions are present in both structures [minimum C⋯ring-centroid separations = 3.630 (2) and 3.581 (2) Å, respectively].

## Abstract 2

In the crystal structures of the racemic *para* and *ortho* isomers of 2-(4-nitro­phen­yl)-3-phenyl-2,3-di­hydro-4*H*-1,3-benzo­thia­zin-4-one, the six-membered thia­zine ring of the benzo­thia­zone parent mol­ecule displays a screw-boat conformation and a near-screw-boat conformation, respectively. In the crystals of both isomers, weak C—H⋯O hydrogen-bonding inter­actions give rise to one-dimensional structures.

## Chemical context   

In earlier reports, we described the T3P-promoted synthesis and crystal structures of 2-(3-nitro­phen­yl)-3-phenyl-2,3-di­hydro-4*H*-1,3-benzo­thia­zin-4-one (III) (Yennawar *et al.*, 2013 ▸) and 2,3-diphenyl-2,3-di­hydro-4*H*-1,3-benzo­thia­zin-4-one (IV) (Yennawar *et al.*, 2014 ▸). In compound (III), the phenyl ring substituent on the 2-position of the thia­zinone ring has a nitro group in the *meta* position. 
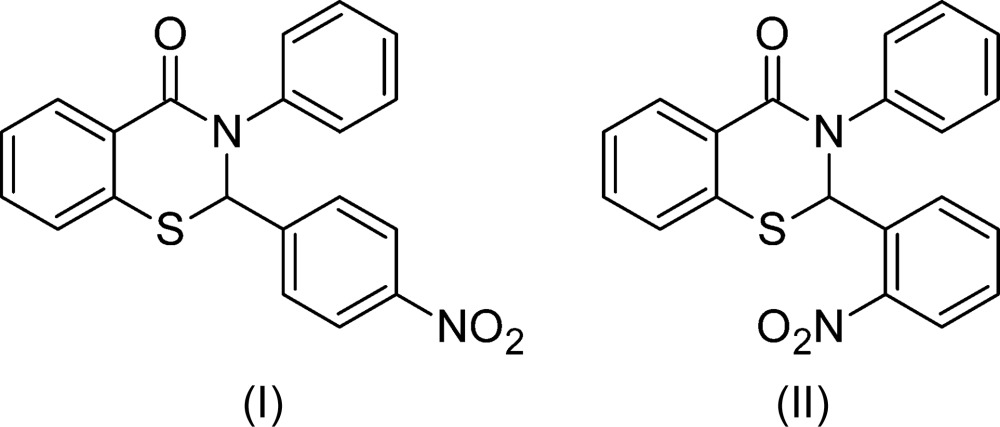


Here we report the synthesis and crystal structures of the *para*- and *ortho*-nitro analogs of C_20_H_14_N_2_O_3_S, the title compounds, 2-(4-nitro­phen­yl)-3-phenyl-2,3-di­hydro-4*H*-1,3-benzo­thia­zin-4-one, (I) and (II), respectively, completing the set and allowing for comparison of the structural effects of the differently positioned nitro substituent groups.

## Structural commentary   

**Figure 1:**
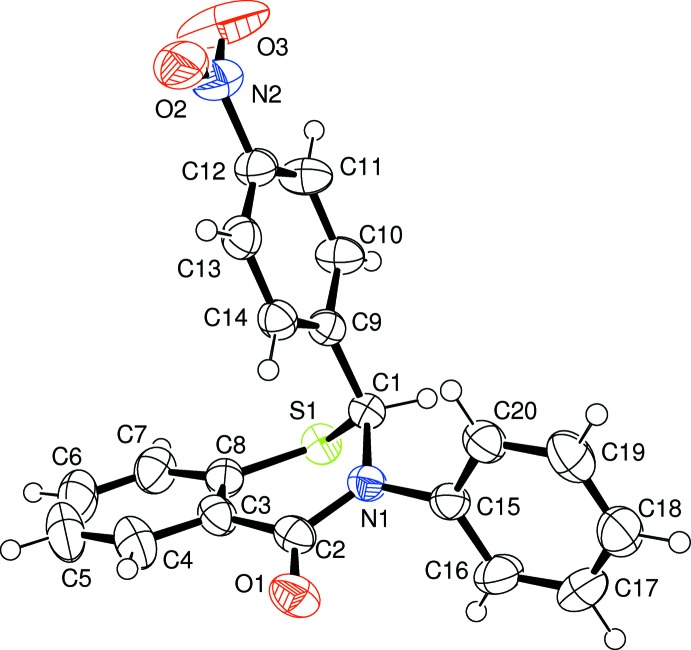
Mol­ecular conformation and atom-numbering scheme for (I). Displacement ellipsoids are drawn at the 50% probability level

**Figure 2:**
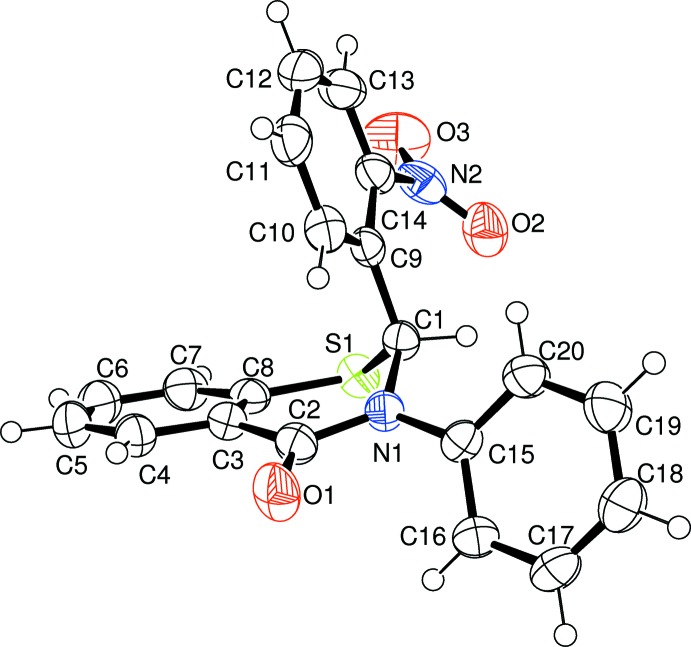
Mol­ecular conformation and atom-numbering scheme for (II). Displacement ellipsoids are drawn at the 50% probability level.

The crystal structures of the two racemic isomers (I) and (II) show some differences and some similarities among themselves as well as with the *meta*-form (III) reported earlier (Yennawar *et al.*, 2013 ▸). The *para*-nitro form (I) (Fig. 1 ▸) is triclinic, space group *P* 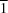, while the *ortho*-nitro form (II) (Fig. 2 ▸) is monoclinic, space group *P*2_1_/*n*, as was the *meta*-form. The structures show screw-boat (I) or near screw-boat (II) conformations for the thia­zine ring, as compared to an envelope conformation in the *meta*-form (III) and the unsubstituted 2,3-diphenyl compound (IV). In both (I) and (II), the three phenyl-ring planes are close to orthogonal with each other, with dihedral angles between the planes of the two substituent groups (C9–C14 = 4-nitrophenyl ring and C15–C20 = phenyl ring) with the benzene ring (C3–C8) of the parent benzo­thia­zine moiety of 75.93 (5) and 82.61 (5)° in (I), and 76.79 (6) and 71.66 (6)° in (II), compared with 81.33 (15) and 75.73 (15)° in the *meta*-isomer (III) and 76.96 (5) and 88.99 (6)° in the unsubstituted 2,3-diphenyl compound (IV) (Yennawar *et al.*, 2014 ▸).

## Supra­molecular features   

**Figure 3:**
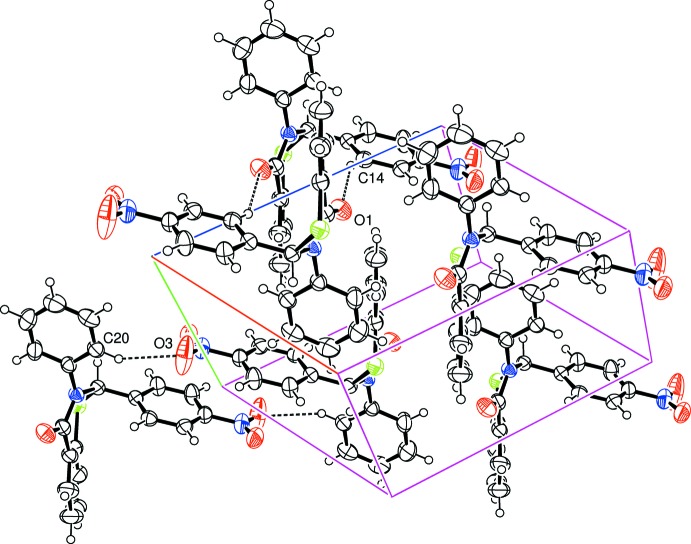
Crystal packing in (I) showing inter­molecular hydrogen-bonding inter­actions as dashed lines.

**Figure 4:**
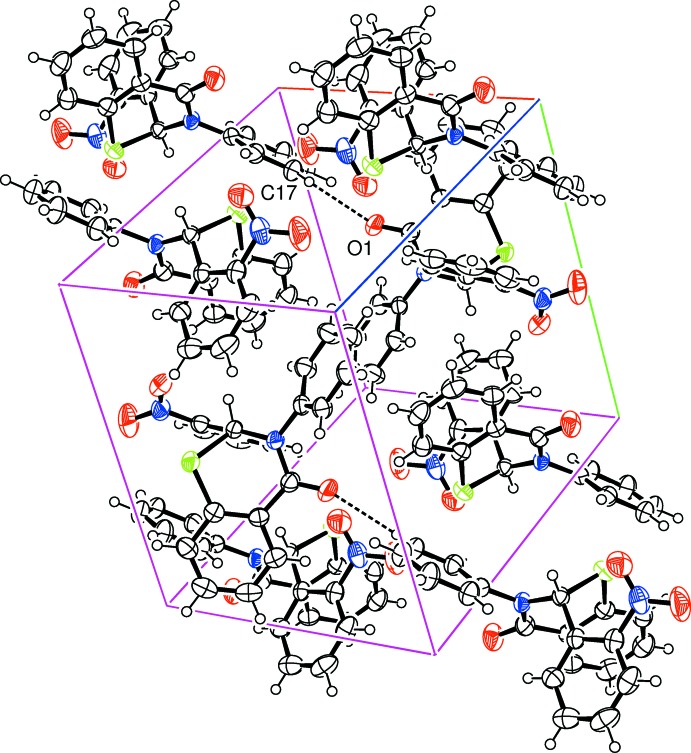
Crystal packing in (II) showing inter­molecular hydrogen-bonding inter­actions as dashed lines.

In (I), as in the *meta*-form (Yennawar *et al.*, 2013 ▸), one of the O atoms of the nitro group accepts a weak aromatic C20—H20⋯O3^i^ hydrogen bond (Table 1 ▸), forming a large centrosymmetric cyclic dimer through an 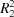
(22) association. A further set of weak inversion-related C14—H14⋯O1^ii^ inter­actions with carbonyl O-atom acceptors give a second cyclic dimer [graph set 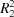
(14)], forming a zigzag chain structure extending along *c* (Fig. 3 ▸). In (II), a weak inter­molecular C17—H17⋯O1^iii^ hydrogen bond to the thia­zinone O-atom acceptor (Table 2 ▸) gives rise to a chain extending along the *b*-axis direction (Fig. 4 ▸). In addition, C—H⋯π inter­actions are present in both (I) (Table 1 ▸) and (II) (Table 2 ▸) [minimum C⋯ring-centroid separations of 3.630 (2) and 3.581 (2) Å, respectively], linking the chains to form sheets in the *bc* plane in (I) and a three-dimensional structure in (II). There are no other significant interactions present in either structure.

## Database survey   

Along with 2-(3-nitro­phen­yl)-3-phenyl-2,3-di­hydro-4*H*-1,3-benzo­thia­zin-4-one (Yennawar *et al.*, 2013 ▸), we have also previously reported the structure of the non-nitro-substituted analog 2,3-diphenyl-2,3-di­hydro-4*H*-1,3-benzo­thia­zin-4-one (Yennawar *et al.*, 2014 ▸).

## Synthesis and crystallization   

The syntheses were achieved in the manner previously reported, by condensation of thio­salicylic acid with a diaryl imine (Yennawar *et al.*, 2013 ▸, 2014 ▸), as follows:

A two-necked 25 ml round-bottomed flask was oven-dried, cooled under N_2_, and charged with a stir bar and the imine (6 mmol). Tetra­hydro­furan (2.3 ml) was added, the solid dissolved, and the solution was stirred. Pyridine (1.95 ml, 24 mmol) was added after which thio­salicylic acid (0.93 g, 6 mmol) was added. Finally, 2,4,6-tripropyl-1,3,5,2,4,6-trioxa­tri­phospho­rinane 2,4,6-trioxide (T3P) in 2-methyl­tetra­hydro­furan (50% *w*/*w*; 7.3 ml, 12 mmol) was added. The reaction was stirred at room temperature and followed by TLC. The mixture was poured into a separatory funnel with di­chloro­methane and distilled water. The layers were separated and the aqueous fraction was then extracted twice with di­chloro­methane. The organic fractions were combined and washed with saturated aqueous solutions of sodium bicarbonate and then saturated sodium chloride. The organic fraction was dried over sodium sulfate and concentrated under vacuum. The crude solid was chromatographed on 30 g flash silica gel and then recrystallized as described below.

(I): 2-(4-Nitro­phen­yl)-3-phenyl-2,3-di­hydro-4*H*-1,3-benzo­thia­zin-4-one: Recrystallized twice, first from ethanol and then from hexa­nes. Yield: 0.162 g (7.4%); m.p. 453–456 K. *R_f_* = 0.55 (40% ethyl acetate/hexa­nes). Crystals for X-ray crystallography were grown by slow evaporation from ethanol.

(II): 2-(2-Nitro­phen­yl)-3-phenyl-2,3-di­hydro-4*H*-1,3-benzothia­zin-4-one: Recrystallized from ethanol. Yield: 0.301 g (13.8%); m.p. 445–450 K. *R_f_* = 0.33 (30% ethyl acetate/hexa­nes). Crystals for X-ray crystallography were grown by slow evaporation from ethyl acetate.

## Refinement details   

Crystal data, data collection and structure refinement details for structures (I) and (II) are summarized in Table 3 ▸. The H atoms were placed geometrically, with C—H = 0.93–0.97 Å, and refined as riding, with *U*_iso_(H) = 1.2*U*_eq_(C).

## Supplementary Material

Crystal structure: contains datablock(s) I, II, 1. DOI: 10.1107/S2056989015004545/zs2326sup1.cif

Structure factors: contains datablock(s) I. DOI: 10.1107/S2056989015004545/zs2326Isup4.hkl

Structure factors: contains datablock(s) II. DOI: 10.1107/S2056989015004545/zs2326IIsup5.hkl

Supporting information file. DOI: 10.1107/S2056989015004545/zs2326Isup4.cml

Supporting information file. DOI: 10.1107/S2056989015004545/zs2326IIsup5.cml

CCDC references: 1052205, 1052204

Additional supporting information:  crystallographic information; 3D view; checkCIF report

## Figures and Tables

**Table 1:** Hydrogen-bond geometry (Å, °) for (I) *Cg*1 and *Cg*2 are the centroids of the phenyl rings C15–C20 and C3–C8, respectively.

*D*—H⋯*A*	*D*—H	H⋯*A*	*D*⋯*A*	*D*—H⋯*A*
C20—H20⋯O3^i^	0.93	2.68	3.468 (2)	143
C14—H14⋯O1^ii^	0.93	2.65	3.4886 (17)	150
C11—H11⋯*Cg*1^iii^	0.93	2.85	3.646 (2)	144
C17—H17⋯*Cg*2^iv^	0.93	2.77	3.630 (2)	154

Symmetry codes: (i) 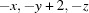; (ii) 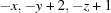; (iii) 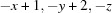; (iv) 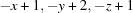.

**Table 2:** Hydrogen-bond geometry (Å, °) for (II) *Cg*3 is the centroid of the C15–C20 ring.

*D*—H⋯*A*	*D*—H	H⋯*A*	*D*⋯*A*	*D*—H⋯*A*
C17—H17⋯O1^v^	0.93	2.58	3.234 (2)	128
C6—H6⋯*Cg*3^vi^	0.93	2.68	3.581 (2)	163

Symmetry codes: (v) 
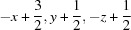
; (vi) 
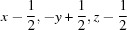
.

**Table 3:** Experimental details

	(I)	(II)
Crystal data		
Chemical formula	C_20_H_14_N_2_O_3_S	C_20_H_14_N_2_O_3_S
*M*_r_	362.39	362.39
Crystal system, space group	Triclinic, *P* 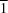	Monoclinic, *P*2_1_/*n*
Temperature (K)	298	298
*a*, *b*, *c* (Å)	8.1787 (12), 9.6190 (14), 12.0881 (18)	10.7396 (19), 11.778 (2), 13.532 (2)
α, β, γ (°)	73.673 (3), 71.158 (3), 86.167 (3)	90, 96.933 (3), 90
*V* (Å^3^)	863.4 (2)	1699.2 (5)
*Z*	2	4
Radiation type	Mo *K*α	Mo *K*α
μ (mm^−1^)	0.21	0.21
Crystal size (mm)	0.22 × 0.20 × 0.11	0.24 × 0.13 × 0.13

Data collection		
Diffractometer	Bruker SMART CCD area detector	Bruker CCD area detector
Absorption correction	Multi-scan (*SADABS*; Bruker, 2001 ▸)	Multi-scan (*SADABS*; Bruker, 2001 ▸)
*T*_min_, *T*_max_	0.944, 0.980	0.951, 0.973
No. of measured, independent and observed [*I* > 2σ(*I*)] reflections	6717, 4134, 3740	15447, 4192, 3307
*R*_int_	0.011	0.027
(sin θ/λ)_max_ (Å^−1^)	0.667	0.667

Refinement		
*R*[*F* ^2^ > 2σ(*F* ^2^)], *wR*(*F* ^2^), *S*	0.040, 0.114, 1.04	0.051, 0.128, 1.03
No. of reflections	4134	4192
No. of parameters	235	235
H-atom treatment	H-atom parameters constrained	H-atom parameters not refined
Δρ_max_, Δρ_min_ (e Å^−3^)	0.27, −0.22	0.32, −0.24

Computer programs: *SMART* and *SAINT* (Bruker, 2001 ▸), *SHELXS97*, *SHELXL97* and *SHELXTL* (Sheldrick, 2008 ▸) and *PLATON* (Spek, 2009 ▸).

## supplementary crystallographic information

### (I) 2-(4-Nitrophenyl)-3-phenyl-2,3-dihydro-4H-1,3-benzothiazin-4-one Crystal data

**Table:**

C_20_H_14_N_2_O_3_S	*Z* = 2
*M**_r_* = 362.39	*F*(000) = 376
Triclinic, *P*1	*D*_x_ = 1.394 Mg m^−^^3^
Hall symbol: -P 1	Melting point = 453–456 K
*a* = 8.1787 (12) Å	Mo *K*α radiation, λ = 0.71073 Å
*b* = 9.6190 (14) Å	Cell parameters from 4358 reflections
*c* = 12.0881 (18) Å	θ = 2.5–28.3°
α = 73.673 (3)°	µ = 0.21 mm^−^^1^
β = 71.158 (3)°	*T* = 298 K
γ = 86.167 (3)°	Block, colorless
*V* = 863.4 (2) Å^3^	0.22 × 0.20 × 0.11 mm

### (I) 2-(4-Nitrophenyl)-3-phenyl-2,3-dihydro-4H-1,3-benzothiazin-4-one Data collection

**Table:**

Bruker SMART CCD area-detector diffractometer	4134 independent reflections
Radiation source: fine-focus sealed tube	3740 reflections with *I* > 2σ(*I*)
Graphite monochromator	*R*_int_ = 0.011
Detector resolution: 8.34 pixels mm^-1^	θ_max_ = 28.3°, θ_min_ = 1.9°
φ and ω scans	*h* = −10→10
Absorption correction: multi-scan (SADABS;Bruker, 2001)	*k* = −12→12
*T*_min_ = 0.944, *T*_max_ = 0.980	*l* = −15→16
6717 measured reflections	

### (I) 2-(4-Nitrophenyl)-3-phenyl-2,3-dihydro-4H-1,3-benzothiazin-4-one Refinement

**Table:**

Refinement on *F*^2^	Primary atom site location: structure-invariant direct methods
Least-squares matrix: full	Secondary atom site location: difference Fourier map
*R*[*F*^2^ > 2σ(*F*^2^)] = 0.040	Hydrogen site location: inferred from neighbouring sites
*wR*(*F*^2^) = 0.114	H-atom parameters constrained
*S* = 1.04	*w* = 1/[σ^2^(*F*_o_^2^) + (0.0641*P*)^2^ + 0.1897*P*] where *P* = (*F*_o_^2^ + 2*F*_c_^2^)/3
4134 reflections	(Δ/σ)_max_ < 0.001
235 parameters	Δρ_max_ = 0.27 e Å^−^^3^
0 restraints	Δρ_min_ = −0.22 e Å^−^^3^

### (I) 2-(4-Nitrophenyl)-3-phenyl-2,3-dihydro-4H-1,3-benzothiazin-4-one Special details

**Table:**

Geometry. All e.s.d.'s (except the e.s.d. in the dihedral angle between two l.s. planes) are estimated using the full covariance matrix. The cell e.s.d.'s are taken into account individually in the estimation of e.s.d.'s in distances, angles and torsion angles; correlations between e.s.d.'s in cell parameters are only used when they are defined by crystal symmetry. An approximate (isotropic) treatment of cell e.s.d.'s is used for estimating e.s.d.'s involving l.s. planes.
Refinement. Refinement of *F*^2^ against ALL reflections. The weighted *R*-factor *wR* and goodness of fit *S* are based on *F*^2^, conventional *R*-factors *R* are based on *F*, with *F* set to zero for negative *F*^2^. The threshold expression of *F*^2^ > σ(*F*^2^) is used only for calculating *R*-factors(gt) *etc*. and is not relevant to the choice of reflections for refinement. *R*-factors based on *F*^2^ are statistically about twice as large as those based on *F*, and *R*- factors based on ALL data will be even larger.

### (I) 2-(4-Nitrophenyl)-3-phenyl-2,3-dihydro-4H-1,3-benzothiazin-4-one Fractional atomic coordinates and isotropic or equivalent isotropic displacement parameters (Å^2^)

**Table:**

	*x*	*y*	*z*	*U*_iso_*/*U*_eq_	
C1	0.40466 (16)	0.89852 (13)	0.24333 (11)	0.0341 (2)	
H1	0.4994	0.9538	0.1750	0.041*	
C2	0.24767 (17)	0.93163 (15)	0.44754 (11)	0.0391 (3)	
C3	0.20773 (17)	0.77262 (15)	0.49057 (12)	0.0410 (3)	
C4	0.0634 (2)	0.7216 (2)	0.59206 (15)	0.0564 (4)	
H4	−0.0095	0.7869	0.6269	0.068*	
C5	0.0280 (2)	0.5748 (2)	0.64110 (17)	0.0680 (5)	
H5	−0.0695	0.5418	0.7081	0.082*	
C6	0.1362 (3)	0.47728 (19)	0.59128 (18)	0.0648 (5)	
H6	0.1130	0.3784	0.6262	0.078*	
C7	0.2786 (2)	0.52435 (16)	0.49030 (16)	0.0524 (4)	
H7	0.3506	0.4577	0.4566	0.063*	
C8	0.31425 (17)	0.67210 (15)	0.43888 (12)	0.0401 (3)	
C9	0.26990 (16)	0.87025 (13)	0.18964 (11)	0.0341 (2)	
C10	0.32579 (18)	0.81159 (17)	0.09111 (13)	0.0453 (3)	
H10	0.4421	0.7916	0.0610	0.054*	
C11	0.2116 (2)	0.78271 (18)	0.03749 (14)	0.0506 (4)	
H11	0.2490	0.7427	−0.0279	0.061*	
C12	0.03947 (18)	0.81485 (15)	0.08353 (13)	0.0426 (3)	
C13	−0.01978 (17)	0.87506 (15)	0.17920 (12)	0.0417 (3)	
H13	−0.1357	0.8970	0.2075	0.050*	
C14	0.09643 (17)	0.90258 (14)	0.23300 (12)	0.0382 (3)	
H14	0.0583	0.9428	0.2982	0.046*	
C15	0.40376 (17)	1.13574 (13)	0.28246 (11)	0.0365 (3)	
C16	0.5298 (2)	1.18389 (18)	0.31737 (16)	0.0568 (4)	
H16	0.5749	1.1211	0.3736	0.068*	
C17	0.5879 (3)	1.3269 (2)	0.26738 (18)	0.0690 (5)	
H17	0.6711	1.3606	0.2915	0.083*	
C18	0.5242 (3)	1.41946 (17)	0.18278 (15)	0.0614 (4)	
H18	0.5650	1.5150	0.1492	0.074*	
C19	0.4004 (2)	1.37082 (17)	0.14789 (14)	0.0547 (4)	
H19	0.3571	1.4336	0.0906	0.066*	
C20	0.33929 (19)	1.22879 (16)	0.19738 (12)	0.0440 (3)	
H20	0.2551	1.1961	0.1735	0.053*	
N1	0.34692 (14)	0.98628 (11)	0.32873 (9)	0.0368 (2)	
N2	−0.08292 (19)	0.78339 (17)	0.02700 (13)	0.0568 (3)	
O1	0.19567 (15)	1.00925 (12)	0.51528 (9)	0.0529 (3)	
O2	−0.22860 (15)	0.82895 (17)	0.05535 (12)	0.0707 (4)	
O3	−0.0330 (2)	0.7167 (3)	−0.0480 (2)	0.1181 (8)	
S1	0.49691 (4)	0.73128 (4)	0.31028 (3)	0.04094 (11)	

### (I) 2-(4-Nitrophenyl)-3-phenyl-2,3-dihydro-4H-1,3-benzothiazin-4-one Atomic displacement parameters (Å^2^)

**Table:**

	*U*^11^	*U*^22^	*U*^33^	*U*^12^	*U*^13^	*U*^23^
C1	0.0348 (6)	0.0353 (6)	0.0318 (6)	0.0012 (5)	−0.0101 (5)	−0.0094 (5)
C2	0.0393 (6)	0.0450 (7)	0.0332 (6)	0.0048 (5)	−0.0138 (5)	−0.0093 (5)
C3	0.0388 (6)	0.0456 (7)	0.0347 (6)	0.0016 (5)	−0.0123 (5)	−0.0041 (5)
C4	0.0453 (8)	0.0621 (9)	0.0467 (8)	0.0013 (7)	−0.0056 (6)	−0.0021 (7)
C5	0.0507 (9)	0.0703 (11)	0.0587 (10)	−0.0111 (8)	−0.0054 (8)	0.0090 (9)
C6	0.0646 (10)	0.0487 (9)	0.0688 (11)	−0.0133 (8)	−0.0243 (9)	0.0098 (8)
C7	0.0548 (9)	0.0413 (7)	0.0595 (9)	−0.0001 (6)	−0.0244 (7)	−0.0035 (6)
C8	0.0381 (6)	0.0409 (7)	0.0405 (7)	0.0000 (5)	−0.0170 (5)	−0.0046 (5)
C9	0.0367 (6)	0.0336 (6)	0.0319 (6)	0.0023 (5)	−0.0120 (5)	−0.0079 (5)
C10	0.0407 (7)	0.0593 (8)	0.0430 (7)	0.0136 (6)	−0.0167 (6)	−0.0241 (6)
C11	0.0530 (8)	0.0646 (9)	0.0479 (8)	0.0154 (7)	−0.0239 (7)	−0.0306 (7)
C12	0.0445 (7)	0.0454 (7)	0.0425 (7)	0.0000 (6)	−0.0212 (6)	−0.0102 (6)
C13	0.0340 (6)	0.0467 (7)	0.0407 (7)	−0.0005 (5)	−0.0102 (5)	−0.0079 (6)
C14	0.0374 (6)	0.0417 (6)	0.0340 (6)	0.0014 (5)	−0.0080 (5)	−0.0118 (5)
C15	0.0420 (6)	0.0358 (6)	0.0334 (6)	0.0015 (5)	−0.0128 (5)	−0.0115 (5)
C16	0.0738 (11)	0.0524 (8)	0.0566 (9)	−0.0065 (8)	−0.0390 (8)	−0.0114 (7)
C17	0.0899 (13)	0.0618 (10)	0.0694 (11)	−0.0207 (9)	−0.0350 (10)	−0.0233 (9)
C18	0.0889 (13)	0.0397 (7)	0.0505 (9)	−0.0095 (8)	−0.0105 (8)	−0.0156 (7)
C19	0.0682 (10)	0.0441 (8)	0.0421 (8)	0.0071 (7)	−0.0120 (7)	−0.0044 (6)
C20	0.0447 (7)	0.0478 (7)	0.0372 (7)	0.0013 (6)	−0.0141 (6)	−0.0070 (6)
N1	0.0433 (6)	0.0359 (5)	0.0319 (5)	−0.0005 (4)	−0.0117 (4)	−0.0104 (4)
N2	0.0553 (8)	0.0679 (9)	0.0579 (8)	0.0003 (6)	−0.0305 (7)	−0.0190 (7)
O1	0.0624 (7)	0.0565 (6)	0.0385 (5)	0.0077 (5)	−0.0104 (5)	−0.0191 (5)
O2	0.0425 (6)	0.1119 (11)	0.0615 (7)	−0.0054 (6)	−0.0213 (5)	−0.0225 (7)
O3	0.1007 (13)	0.1719 (19)	0.1604 (18)	0.0508 (12)	−0.0875 (13)	−0.1248 (17)
S1	0.03605 (18)	0.04201 (19)	0.04357 (19)	0.00672 (13)	−0.01346 (14)	−0.01027 (14)

### (I) 2-(4-Nitrophenyl)-3-phenyl-2,3-dihydro-4H-1,3-benzothiazin-4-one Geometric parameters (Å, º)

**Table:**

C1—N1	1.4570 (15)	C11—C12	1.386 (2)
C1—C9	1.5203 (17)	C11—H11	0.9300
C1—S1	1.8200 (13)	C12—C13	1.373 (2)
C1—H1	0.9800	C12—N2	1.4695 (18)
C2—O1	1.2186 (17)	C13—C14	1.3878 (19)
C2—N1	1.3702 (17)	C13—H13	0.9300
C2—C3	1.4921 (19)	C14—H14	0.9300
C3—C4	1.394 (2)	C15—C20	1.3807 (19)
C3—C8	1.400 (2)	C15—C16	1.3817 (19)
C4—C5	1.379 (3)	C15—N1	1.4371 (16)
C4—H4	0.9300	C16—C17	1.385 (2)
C5—C6	1.374 (3)	C16—H16	0.9300
C5—H5	0.9300	C17—C18	1.371 (3)
C6—C7	1.376 (3)	C17—H17	0.9300
C6—H6	0.9300	C18—C19	1.368 (3)
C7—C8	1.390 (2)	C18—H18	0.9300
C7—H7	0.9300	C19—C20	1.383 (2)
C8—S1	1.7574 (14)	C19—H19	0.9300
C9—C14	1.3916 (18)	C20—H20	0.9300
C9—C10	1.3920 (18)	N2—O3	1.205 (2)
C10—C11	1.377 (2)	N2—O2	1.2166 (19)
C10—H10	0.9300		
			
N1—C1—C9	114.94 (10)	C12—C11—H11	120.9
N1—C1—S1	110.58 (8)	C13—C12—C11	122.37 (12)
C9—C1—S1	111.83 (8)	C13—C12—N2	119.26 (13)
N1—C1—H1	106.3	C11—C12—N2	118.37 (13)
C9—C1—H1	106.3	C12—C13—C14	118.81 (13)
S1—C1—H1	106.3	C12—C13—H13	120.6
O1—C2—N1	121.47 (13)	C14—C13—H13	120.6
O1—C2—C3	121.52 (12)	C13—C14—C9	120.22 (12)
N1—C2—C3	117.00 (11)	C13—C14—H14	119.9
C4—C3—C8	118.67 (14)	C9—C14—H14	119.9
C4—C3—C2	118.08 (13)	C20—C15—C16	120.19 (13)
C8—C3—C2	123.11 (12)	C20—C15—N1	119.55 (12)
C5—C4—C3	120.44 (17)	C16—C15—N1	120.15 (12)
C5—C4—H4	119.8	C15—C16—C17	119.06 (15)
C3—C4—H4	119.8	C15—C16—H16	120.5
C6—C5—C4	120.23 (16)	C17—C16—H16	120.5
C6—C5—H5	119.9	C18—C17—C16	120.85 (16)
C4—C5—H5	119.9	C18—C17—H17	119.6
C5—C6—C7	120.67 (16)	C16—C17—H17	119.6
C5—C6—H6	119.7	C19—C18—C17	119.80 (15)
C7—C6—H6	119.7	C19—C18—H18	120.1
C6—C7—C8	119.64 (16)	C17—C18—H18	120.1
C6—C7—H7	120.2	C18—C19—C20	120.37 (15)
C8—C7—H7	120.2	C18—C19—H19	119.8
C7—C8—C3	120.31 (14)	C20—C19—H19	119.8
C7—C8—S1	119.34 (12)	C15—C20—C19	119.72 (14)
C3—C8—S1	120.34 (10)	C15—C20—H20	120.1
C14—C9—C10	119.35 (12)	C19—C20—H20	120.1
C14—C9—C1	123.12 (11)	C2—N1—C15	120.70 (10)
C10—C9—C1	117.53 (11)	C2—N1—C1	123.15 (11)
C11—C10—C9	121.02 (13)	C15—N1—C1	116.13 (10)
C11—C10—H10	119.5	O3—N2—O2	122.84 (14)
C9—C10—H10	119.5	O3—N2—C12	118.39 (15)
C10—C11—C12	118.22 (13)	O2—N2—C12	118.73 (14)
C10—C11—H11	120.9	C8—S1—C1	96.00 (6)
			
O1—C2—C3—C4	22.9 (2)	C20—C15—C16—C17	1.1 (3)
N1—C2—C3—C4	−157.74 (13)	N1—C15—C16—C17	177.15 (16)
O1—C2—C3—C8	−152.79 (14)	C15—C16—C17—C18	−1.2 (3)
N1—C2—C3—C8	26.57 (18)	C16—C17—C18—C19	0.7 (3)
C8—C3—C4—C5	0.8 (2)	C17—C18—C19—C20	−0.1 (3)
C2—C3—C4—C5	−175.10 (15)	C16—C15—C20—C19	−0.5 (2)
C3—C4—C5—C6	0.9 (3)	N1—C15—C20—C19	−176.61 (13)
C4—C5—C6—C7	−1.6 (3)	C18—C19—C20—C15	0.0 (2)
C5—C6—C7—C8	0.6 (3)	O1—C2—N1—C15	4.6 (2)
C6—C7—C8—C3	1.1 (2)	C3—C2—N1—C15	−174.72 (11)
C6—C7—C8—S1	179.73 (13)	O1—C2—N1—C1	−176.96 (12)
C4—C3—C8—C7	−1.8 (2)	C3—C2—N1—C1	3.67 (18)
C2—C3—C8—C7	173.91 (13)	C20—C15—N1—C2	−112.20 (14)
C4—C3—C8—S1	179.59 (11)	C16—C15—N1—C2	71.69 (18)
C2—C3—C8—S1	−4.74 (18)	C20—C15—N1—C1	69.30 (16)
N1—C1—C9—C14	−10.83 (17)	C16—C15—N1—C1	−106.80 (15)
S1—C1—C9—C14	116.32 (12)	C9—C1—N1—C2	79.64 (15)
N1—C1—C9—C10	167.99 (12)	S1—C1—N1—C2	−48.15 (14)
S1—C1—C9—C10	−64.86 (14)	C9—C1—N1—C15	−101.90 (12)
C14—C9—C10—C11	−1.2 (2)	S1—C1—N1—C15	130.31 (10)
C1—C9—C10—C11	179.93 (13)	C13—C12—N2—O3	−172.21 (19)
C9—C10—C11—C12	0.6 (2)	C11—C12—N2—O3	8.0 (3)
C10—C11—C12—C13	0.6 (2)	C13—C12—N2—O2	9.7 (2)
C10—C11—C12—N2	−179.65 (14)	C11—C12—N2—O2	−170.08 (16)
C11—C12—C13—C14	−1.1 (2)	C7—C8—S1—C1	148.50 (12)
N2—C12—C13—C14	179.16 (12)	C3—C8—S1—C1	−32.84 (12)
C12—C13—C14—C9	0.4 (2)	N1—C1—S1—C8	56.20 (9)
C10—C9—C14—C13	0.7 (2)	C9—C1—S1—C8	−73.27 (9)
C1—C9—C14—C13	179.49 (12)		

### (I) 2-(4-Nitrophenyl)-3-phenyl-2,3-dihydro-4H-1,3-benzothiazin-4-one Hydrogen-bond geometry (Å, º)

Cg1 and Cg2 are the centroids of the phenyl rings C15–C20 and C3–C8, respectively.

**Table:**

*D*—H···*A*	*D*—H	H···*A*	*D*···*A*	*D*—H···*A*
C20—H20···O3^i^	0.93	2.68	3.468 (2)	143
C1—H1···O2^ii^	0.98	2.65	3.2851 (18)	123
C14—H14···O1^iii^	0.93	2.65	3.4886 (17)	150
C11—H11···*Cg*1^iv^	0.93	2.85	3.646 (2)	144
C17—H17···*Cg*2^v^	0.93	2.77	3.630 (2)	154

Symmetry codes: (i) −*x*, −*y*+2, −*z*; (ii) *x*+1, *y*, *z*; (iii) −*x*, −*y*+2, −*z*+1; (iv) −*x*+1, −*y*+2, −*z*; (v) −*x*+1, −*y*+2, −*z*+1.

### (II) 2-(2-Nitrophenyl)-3-phenyl-2,3-dihydro-4H-1,3-benzothiazin-4-one Crystal data

**Table:**

C_20_H_14_N_2_O_3_S	*F*(000) = 752
*M**_r_* = 362.39	*D*_x_ = 1.417 Mg m^−^^3^
Monoclinic, *P*2_1_/*n*	Melting point = 445–450 K
Hall symbol: -P 2yn	Mo *K*α radiation, λ = 0.71073 Å
*a* = 10.7396 (19) Å	Cell parameters from 4222 reflections
*b* = 11.778 (2) Å	θ = 2.3–28.2°
*c* = 13.532 (2) Å	µ = 0.21 mm^−^^1^
β = 96.933 (3)°	*T* = 298 K
*V* = 1699.2 (5) Å^3^	Block, colorless
*Z* = 4	0.24 × 0.13 × 0.13 mm

### (II) 2-(2-Nitrophenyl)-3-phenyl-2,3-dihydro-4H-1,3-benzothiazin-4-one Data collection

**Table:**

Bruker CCD area-detector diffractometer	4192 independent reflections
Radiation source: fine-focus sealed tube	3307 reflections with *I* > 2σ(*I*)
Graphite monochromator	*R*_int_ = 0.027
Detector resolution: 8.34 pixels mm^-1^	θ_max_ = 28.3°, θ_min_ = 2.3°
φ and ω scans	*h* = −14→14
Absorption correction: multi-scan (SADABS; Bruker, 2001)	*k* = −15→14
*T*_min_ = 0.951, *T*_max_ = 0.973	*l* = −17→17
15447 measured reflections	

### (II) 2-(2-Nitrophenyl)-3-phenyl-2,3-dihydro-4H-1,3-benzothiazin-4-one Refinement

**Table:**

Refinement on *F*^2^	Primary atom site location: structure-invariant direct methods
Least-squares matrix: full	Secondary atom site location: difference Fourier map
*R*[*F*^2^ > 2σ(*F*^2^)] = 0.051	Hydrogen site location: inferred from neighbouring sites
*wR*(*F*^2^) = 0.128	H-atom parameters not refined
*S* = 1.03	*w* = 1/[σ^2^(*F*_o_^2^) + (0.0634*P*)^2^ + 0.429*P*] where *P* = (*F*_o_^2^ + 2*F*_c_^2^)/3
4192 reflections	(Δ/σ)_max_ < 0.001
235 parameters	Δρ_max_ = 0.32 e Å^−^^3^
0 restraints	Δρ_min_ = −0.24 e Å^−^^3^

### (II) 2-(2-Nitrophenyl)-3-phenyl-2,3-dihydro-4H-1,3-benzothiazin-4-one Special details

**Table:**

Geometry. All e.s.d.'s (except the e.s.d. in the dihedral angle between two l.s. planes) are estimated using the full covariance matrix. The cell e.s.d.'s are taken into account individually in the estimation of e.s.d.'s in distances, angles and torsion angles; correlations between e.s.d.'s in cell parameters are only used when they are defined by crystal symmetry. An approximate (isotropic) treatment of cell e.s.d.'s is used for estimating e.s.d.'s involving l.s. planes.
Refinement. Refinement of *F*^2^ against ALL reflections. The weighted *R*-factor *wR* and goodness of fit *S* are based on *F*^2^, conventional *R*-factors *R* are based on *F*, with *F* set to zero for negative *F*^2^. The threshold expression of *F*^2^ > σ(*F*^2^) is used only for calculating *R*-factors(gt) *etc*. and is not relevant to the choice of reflections for refinement. *R*-factors based on *F*^2^ are statistically about twice as large as those based on *F*, and *R*- factors based on ALL data will be even larger.

### (II) 2-(2-Nitrophenyl)-3-phenyl-2,3-dihydro-4H-1,3-benzothiazin-4-one Fractional atomic coordinates and isotropic or equivalent isotropic displacement parameters (Å^2^)

**Table:**

	*x*	*y*	*z*	*U*_iso_*/*U*_eq_	
C1	0.28981 (14)	0.46215 (14)	0.18506 (13)	0.0331 (4)	
H1	0.2834	0.5426	0.2022	0.040*	
C2	0.46574 (15)	0.33061 (16)	0.16797 (14)	0.0397 (4)	
C3	0.37791 (15)	0.26178 (15)	0.09805 (13)	0.0366 (4)	
C4	0.40908 (18)	0.14818 (16)	0.08454 (14)	0.0436 (4)	
H4	0.4780	0.1169	0.1233	0.052*	
C5	0.3393 (2)	0.08211 (18)	0.01489 (16)	0.0522 (5)	
H5	0.3592	0.0059	0.0082	0.063*	
C6	0.2394 (2)	0.12935 (19)	−0.04536 (16)	0.0533 (5)	
H6	0.1943	0.0853	−0.0942	0.064*	
C7	0.20612 (18)	0.24112 (18)	−0.03363 (14)	0.0468 (5)	
H7	0.1388	0.2723	−0.0744	0.056*	
C8	0.27389 (16)	0.30731 (15)	0.03961 (13)	0.0374 (4)	
C9	0.21338 (14)	0.39669 (14)	0.25355 (12)	0.0335 (4)	
C10	0.27005 (18)	0.31979 (15)	0.32244 (14)	0.0429 (4)	
H10	0.3563	0.3091	0.3270	0.051*	
C11	0.2016 (2)	0.25840 (18)	0.38472 (14)	0.0522 (5)	
H11	0.2424	0.2082	0.4309	0.063*	
C12	0.0733 (2)	0.2715 (2)	0.37837 (16)	0.0591 (6)	
H12	0.0274	0.2296	0.4196	0.071*	
C13	0.0137 (2)	0.3465 (2)	0.31115 (16)	0.0545 (5)	
H13	−0.0729	0.3552	0.3060	0.065*	
C14	0.08287 (16)	0.40890 (17)	0.25134 (13)	0.0402 (4)	
C15	0.50767 (15)	0.51223 (15)	0.25360 (13)	0.0353 (4)	
C16	0.62065 (16)	0.53924 (16)	0.21859 (14)	0.0408 (4)	
H16	0.6429	0.5045	0.1615	0.049*	
C17	0.69952 (17)	0.61808 (18)	0.26934 (15)	0.0474 (5)	
H17	0.7754	0.6358	0.2465	0.057*	
C18	0.66703 (19)	0.67052 (19)	0.35309 (16)	0.0533 (5)	
H18	0.7207	0.7234	0.3868	0.064*	
C19	0.55443 (19)	0.6444 (2)	0.38714 (16)	0.0545 (5)	
H19	0.5319	0.6805	0.4435	0.065*	
C20	0.47462 (17)	0.56458 (17)	0.33784 (15)	0.0455 (5)	
H20	0.3993	0.5465	0.3615	0.055*	
N1	0.42334 (12)	0.43381 (12)	0.19877 (11)	0.0357 (3)	
N2	0.01309 (14)	0.49390 (17)	0.18749 (13)	0.0497 (4)	
O1	0.57182 (12)	0.29780 (12)	0.19525 (12)	0.0591 (4)	
O2	0.06296 (14)	0.58460 (14)	0.17327 (12)	0.0602 (4)	
O3	−0.09447 (14)	0.47116 (19)	0.15352 (15)	0.0854 (6)	
S1	0.22755 (4)	0.44863 (4)	0.05436 (3)	0.04088 (15)	

### (II) 2-(2-Nitrophenyl)-3-phenyl-2,3-dihydro-4H-1,3-benzothiazin-4-one Atomic displacement parameters (Å^2^)

**Table:**

	*U*^11^	*U*^22^	*U*^33^	*U*^12^	*U*^13^	*U*^23^
C1	0.0288 (7)	0.0318 (9)	0.0379 (9)	0.0023 (6)	0.0007 (6)	−0.0011 (7)
C2	0.0333 (8)	0.0393 (10)	0.0466 (10)	0.0038 (7)	0.0054 (7)	−0.0010 (8)
C3	0.0377 (8)	0.0358 (9)	0.0373 (9)	−0.0005 (7)	0.0088 (7)	−0.0009 (7)
C4	0.0498 (10)	0.0381 (10)	0.0446 (10)	0.0029 (8)	0.0120 (8)	0.0015 (8)
C5	0.0664 (13)	0.0383 (11)	0.0544 (12)	−0.0044 (9)	0.0179 (10)	−0.0094 (9)
C6	0.0589 (12)	0.0542 (13)	0.0473 (11)	−0.0127 (10)	0.0090 (9)	−0.0137 (10)
C7	0.0466 (10)	0.0545 (12)	0.0391 (10)	−0.0037 (9)	0.0039 (8)	−0.0051 (9)
C8	0.0383 (8)	0.0416 (10)	0.0339 (9)	−0.0012 (7)	0.0104 (7)	0.0013 (7)
C9	0.0336 (8)	0.0312 (9)	0.0353 (8)	0.0002 (6)	0.0031 (6)	−0.0078 (7)
C10	0.0483 (10)	0.0380 (10)	0.0419 (10)	0.0031 (8)	0.0039 (8)	−0.0018 (8)
C11	0.0805 (14)	0.0378 (11)	0.0394 (10)	−0.0037 (10)	0.0124 (10)	−0.0033 (8)
C12	0.0815 (15)	0.0509 (13)	0.0504 (12)	−0.0203 (11)	0.0305 (11)	−0.0108 (10)
C13	0.0473 (10)	0.0632 (14)	0.0563 (12)	−0.0125 (10)	0.0193 (9)	−0.0170 (11)
C14	0.0364 (8)	0.0436 (10)	0.0408 (10)	−0.0010 (7)	0.0056 (7)	−0.0112 (8)
C15	0.0299 (7)	0.0355 (9)	0.0392 (9)	−0.0010 (7)	−0.0010 (6)	0.0030 (7)
C16	0.0350 (8)	0.0454 (11)	0.0422 (10)	−0.0016 (7)	0.0050 (7)	0.0013 (8)
C17	0.0337 (8)	0.0512 (12)	0.0565 (12)	−0.0090 (8)	0.0028 (8)	0.0037 (9)
C18	0.0478 (11)	0.0530 (13)	0.0565 (12)	−0.0126 (9)	−0.0045 (9)	−0.0073 (10)
C19	0.0520 (11)	0.0595 (13)	0.0516 (12)	−0.0069 (10)	0.0049 (9)	−0.0183 (10)
C20	0.0382 (9)	0.0511 (12)	0.0479 (11)	−0.0049 (8)	0.0081 (8)	−0.0060 (9)
N1	0.0272 (6)	0.0349 (8)	0.0444 (8)	0.0005 (5)	0.0015 (6)	−0.0035 (6)
N2	0.0339 (8)	0.0658 (12)	0.0491 (10)	0.0129 (8)	0.0040 (7)	−0.0089 (9)
O1	0.0378 (7)	0.0496 (9)	0.0862 (11)	0.0134 (6)	−0.0069 (7)	−0.0107 (8)
O2	0.0572 (9)	0.0487 (9)	0.0732 (11)	0.0146 (7)	0.0014 (7)	−0.0018 (8)
O3	0.0352 (8)	0.1241 (17)	0.0925 (14)	0.0023 (9)	−0.0104 (8)	0.0063 (12)
S1	0.0424 (2)	0.0428 (3)	0.0361 (2)	0.00758 (19)	−0.00063 (18)	0.00295 (19)

### (II) 2-(2-Nitrophenyl)-3-phenyl-2,3-dihydro-4H-1,3-benzothiazin-4-one Geometric parameters (Å, º)

**Table:**

C1—N1	1.462 (2)	C11—C12	1.379 (3)
C1—C9	1.520 (2)	C11—H11	0.9300
C1—S1	1.8207 (17)	C12—C13	1.370 (3)
C1—H1	0.9800	C12—H12	0.9300
C2—O1	1.217 (2)	C13—C14	1.375 (3)
C2—N1	1.380 (2)	C13—H13	0.9300
C2—C3	1.492 (2)	C14—N2	1.468 (3)
C3—C8	1.396 (2)	C15—C20	1.380 (3)
C3—C4	1.397 (3)	C15—C16	1.392 (2)
C4—C5	1.373 (3)	C15—N1	1.436 (2)
C4—H4	0.9300	C16—C17	1.382 (3)
C5—C6	1.384 (3)	C16—H16	0.9300
C5—H5	0.9300	C17—C18	1.372 (3)
C6—C7	1.378 (3)	C17—H17	0.9300
C6—H6	0.9300	C18—C19	1.380 (3)
C7—C8	1.395 (3)	C18—H18	0.9300
C7—H7	0.9300	C19—C20	1.388 (3)
C8—S1	1.7557 (19)	C19—H19	0.9300
C9—C10	1.387 (2)	C20—H20	0.9300
C9—C14	1.406 (2)	N2—O3	1.220 (2)
C10—C11	1.386 (3)	N2—O2	1.221 (2)
C10—H10	0.9300		
			
N1—C1—C9	113.60 (14)	C10—C11—H11	119.9
N1—C1—S1	110.05 (11)	C13—C12—C11	119.80 (19)
C9—C1—S1	112.70 (11)	C13—C12—H12	120.1
N1—C1—H1	106.7	C11—C12—H12	120.1
C9—C1—H1	106.7	C12—C13—C14	119.52 (19)
S1—C1—H1	106.7	C12—C13—H13	120.2
O1—C2—N1	121.25 (16)	C14—C13—H13	120.2
O1—C2—C3	121.10 (16)	C13—C14—C9	122.74 (19)
N1—C2—C3	117.63 (14)	C13—C14—N2	115.91 (17)
C8—C3—C4	118.82 (17)	C9—C14—N2	121.29 (17)
C8—C3—C2	123.50 (16)	C20—C15—C16	120.06 (16)
C4—C3—C2	117.42 (16)	C20—C15—N1	120.35 (15)
C5—C4—C3	120.90 (19)	C16—C15—N1	119.52 (16)
C5—C4—H4	119.6	C17—C16—C15	119.45 (18)
C3—C4—H4	119.6	C17—C16—H16	120.3
C4—C5—C6	119.78 (19)	C15—C16—H16	120.3
C4—C5—H5	120.1	C18—C17—C16	120.73 (18)
C6—C5—H5	120.1	C18—C17—H17	119.6
C7—C6—C5	120.66 (19)	C16—C17—H17	119.6
C7—C6—H6	119.7	C17—C18—C19	119.73 (18)
C5—C6—H6	119.7	C17—C18—H18	120.1
C6—C7—C8	119.68 (19)	C19—C18—H18	120.1
C6—C7—H7	120.2	C18—C19—C20	120.38 (19)
C8—C7—H7	120.2	C18—C19—H19	119.8
C7—C8—C3	120.08 (17)	C20—C19—H19	119.8
C7—C8—S1	118.68 (14)	C15—C20—C19	119.64 (18)
C3—C8—S1	121.22 (13)	C15—C20—H20	120.2
C10—C9—C14	115.90 (17)	C19—C20—H20	120.2
C10—C9—C1	121.04 (15)	C2—N1—C15	120.81 (13)
C14—C9—C1	123.06 (15)	C2—N1—C1	121.14 (14)
C11—C10—C9	121.84 (19)	C15—N1—C1	117.81 (13)
C11—C10—H10	119.1	O3—N2—O2	123.10 (19)
C9—C10—H10	119.1	O3—N2—C14	117.7 (2)
C12—C11—C10	120.2 (2)	O2—N2—C14	119.16 (15)
C12—C11—H11	119.9	C8—S1—C1	96.74 (8)
			
O1—C2—C3—C8	−158.54 (18)	C1—C9—C14—N2	−5.3 (3)
N1—C2—C3—C8	20.1 (3)	C20—C15—C16—C17	0.5 (3)
O1—C2—C3—C4	15.5 (3)	N1—C15—C16—C17	177.50 (16)
N1—C2—C3—C4	−165.94 (16)	C15—C16—C17—C18	−0.6 (3)
C8—C3—C4—C5	0.2 (3)	C16—C17—C18—C19	0.0 (3)
C2—C3—C4—C5	−174.05 (17)	C17—C18—C19—C20	0.7 (3)
C3—C4—C5—C6	2.3 (3)	C16—C15—C20—C19	0.2 (3)
C4—C5—C6—C7	−2.5 (3)	N1—C15—C20—C19	−176.78 (18)
C5—C6—C7—C8	0.1 (3)	C18—C19—C20—C15	−0.8 (3)
C6—C7—C8—C3	2.5 (3)	O1—C2—N1—C15	8.0 (3)
C6—C7—C8—S1	−178.84 (15)	C3—C2—N1—C15	−170.55 (15)
C4—C3—C8—C7	−2.7 (3)	O1—C2—N1—C1	−166.08 (17)
C2—C3—C8—C7	171.27 (16)	C3—C2—N1—C1	15.3 (2)
C4—C3—C8—S1	178.72 (13)	C20—C15—N1—C2	−133.66 (19)
C2—C3—C8—S1	−7.3 (2)	C16—C15—N1—C2	49.4 (2)
N1—C1—C9—C10	1.1 (2)	C20—C15—N1—C1	40.7 (2)
S1—C1—C9—C10	127.10 (15)	C16—C15—N1—C1	−136.33 (17)
N1—C1—C9—C14	−178.54 (15)	C9—C1—N1—C2	71.6 (2)
S1—C1—C9—C14	−52.5 (2)	S1—C1—N1—C2	−55.81 (19)
C14—C9—C10—C11	0.4 (3)	C9—C1—N1—C15	−102.67 (17)
C1—C9—C10—C11	−179.25 (16)	S1—C1—N1—C15	129.90 (13)
C9—C10—C11—C12	1.0 (3)	C13—C14—N2—O3	−35.0 (3)
C10—C11—C12—C13	−0.8 (3)	C9—C14—N2—O3	147.80 (19)
C11—C12—C13—C14	−0.7 (3)	C13—C14—N2—O2	142.80 (19)
C12—C13—C14—C9	2.1 (3)	C9—C14—N2—O2	−34.4 (3)
C12—C13—C14—N2	−175.09 (18)	C7—C8—S1—C1	153.79 (14)
C10—C9—C14—C13	−1.9 (3)	C3—C8—S1—C1	−27.57 (16)
C1—C9—C14—C13	177.69 (17)	N1—C1—S1—C8	55.42 (13)
C10—C9—C14—N2	175.12 (16)	C9—C1—S1—C8	−72.50 (13)

### (II) 2-(2-Nitrophenyl)-3-phenyl-2,3-dihydro-4H-1,3-benzothiazin-4-one Hydrogen-bond geometry (Å, º)

Cg3 is the centroid of the C15–C20 ring.

**Table:**

*D*—H···*A*	*D*—H	H···*A*	*D*···*A*	*D*—H···*A*
C17—H17···O1^i^	0.93	2.58	3.234 (2)	128
C6—H6···*Cg*3^ii^	0.93	2.68	3.581 (2)	163

Symmetry codes: (i) −*x*+3/2, *y*+1/2, −*z*+1/2; (ii) *x*−1/2, −*y*+1/2, *z*−1/2.
